# Understanding the Evolutionary Fate of Finite Populations: The Dynamics of Mutational Effects

**DOI:** 10.1371/journal.pbio.0050094

**Published:** 2007-04-03

**Authors:** Olin K Silander, Olivier Tenaillon, Lin Chao

**Affiliations:** 1 Division of Biology, University of California San Diego, La Jolla, California, United States of America; 2 Institute of Integrative Biology, Eidgenössische Technische Hochschule (ETH) Zurich, Zurich, Switzerland; 3 INSERM U722, Faculté de Médecine Xavier Bichat, Paris, France; 4 Université Paris 7, Paris, France; University of Edinburgh, United Kingdom

## Abstract

The most consistent result in more than two decades of experimental evolution is that the fitness of populations adapting to a constant environment does not increase indefinitely, but reaches a plateau. Using experimental evolution with bacteriophage, we show here that the converse is also true. In populations small enough such that drift overwhelms selection and causes fitness to decrease, fitness declines down to a plateau. We demonstrate theoretically that both of these phenomena must be due either to changes in the ratio of beneficial to deleterious mutations, the size of mutational effects, or both. We use mutation accumulation experiments and molecular data from experimental evolution to show that the most significant change in mutational effects is a drastic increase in the rate of beneficial mutation as fitness decreases. In contrast, the size of mutational effects changes little even as organismal fitness changes over several orders of magnitude. These findings have significant implications for the dynamics of adaptation.

## Author Summary

In any population, two factors determine whether the average fitness of individuals will increase (adaptation) or decrease: the size of the population and the distribution of mutational effects (i.e., the relative rates and effect sizes of beneficial and deleterious mutations). Although it is relatively simple to get quantitative information on population size, it is much harder to gain insight into the distribution of mutational effects. Very little information exists on the relative rates of beneficial versus deleterious effects, on the shapes of mutational distributions, or on whether the distributions change over time. Thus, it remains difficult to even speculate whether a given population will adapt over time. Here, we use laboratory evolution of a bacterial virus to quantify the distribution of mutational effects. Our results reveal that the average impact of a mutation is approximately constant with respect to fitness, that most mutations have small effects, and that the rate of beneficial mutation depends on the fitness of the organism. Our study demonstrates the simple, but perhaps underappreciated fact that mutational effects are dynamic. It also proposes and tests an explicit model of adaptation in which organismal fitness specifies both the rate and distribution of deleterious and beneficial mutations, and it presents specific and testable predictions of the circumstances under which populations will adapt.

## Abstract 3

Experimental evolution of bacteriophage reveals that mutational effects are dynamic and dependent on genetic background, thus providing fundamental and testable insights into the nature of adaptation.

## Introduction

The process of adaptation in finite populations is determined by the distribution of mutational effects on fitness and the size of the population. In large populations, beneficial mutations are frequently fixed, whereas deleterious mutations will only be fixed if they are of vanishingly small effect size (approximately the inverse of the effective population size [1]). In small populations, beneficial mutations may be frequently lost due to drift, whereas the same process may lead to the fixation of moderately sized deleterious mutations. Whether fitness increases or decreases in a population of a given size thus depends on the relative rates of beneficial and deleterious mutations, and the effect sizes of such mutations.

An accurate description of the distribution of mutational effects is thus fundamental to understanding how adaptive changes occur in biological populations. Experimental manipulation of organisms in the laboratory (i.e., mutation accumulation [MA]) [2–15] and genomic and molecular analyses [16–23] have indicated that deleterious mutations are more common than beneficial mutations. However, experimental evolution of large populations in the laboratory has clearly demonstrated the existence of beneficial mutations [24], and several studies have shed some light on their rate and the distribution of their effects [25–27]. Recently, comparative studies have allowed considerable insight into the rate of fixation of both beneficial and deleterious mutations [28–31]. These comparative studies have frequently come to different conclusions, and it has not yet been determined whether this is a result of different methods employed, biases in the datasets used, differences in the demography of the populations (e.g., population bottlenecks or inbreeding), differences in the evolutionary pressures on the populations (e.g., changes in organismal ecology), or some other factor. Resolving both the rate and the effect size of beneficial and deleterious mutations thus requires experiments expressly designed for this purpose.

Importantly, the rate and effect size of beneficial and deleterious mutations are not constant. These quantities change if the fitness of the evolving organism changes. One manifestation of this effect is the fitness plateaus reached by populations adapting to constant environments [24,32,33]. The appearance of fitness plateaus in adapting populations requires that either beneficial mutations become more rare relative to deleterious mutations, or that mutations become smaller in effect size (which has a 3-fold effect, increasing the fixation probability of deleterious mutations, decreasing the fixation probability of beneficial mutations, and decreasing adaptive step sizes when substitutions do occur). It has previously been observed that populations starting from the same genotype often reach plateaus at similar levels of fitness [34–36], despite fundamental differences in the genetic paths taken [37]. This suggests that changes in the rate and distribution of mutational effects have less to do with the specific genetic background of an organism than with the fitness that an organism has attained. In other words, history plays less of a role than adaptation in reaching these fitness plateaus [34].

**Figure 1:**
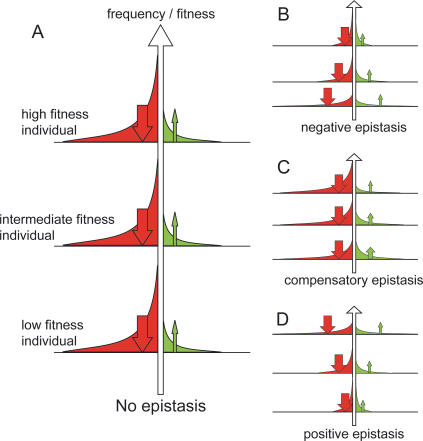
Changes in Mutational Distributions Due to Epistasis The distribution of deleterious mutations appears in red, beneficial mutations in green. Arrows indicate the mean mutational effect; the width of the arrow reflects the rate of deleterious or beneficial mutations. (The total mutation rate remains constant.) Three mutational distributions are illustrated for each epistatic context: individuals of high, intermediate, and low fitness. (A) No epistasis. Mutational distributions are constant regardless of fitness. (B) Negative epistasis. The mean mutational effect increases as fitness decreases. (C) Compensatory epistasis. The mean effect of each mutational class stays constant, whereas the fraction of mutations that are beneficial increases as fitness decreases. (D) Positive epistasis. The mean mutational effect decreases as fitness decreases.

There is an analogous process that occurs in populations that experience decreases in fitness. This process is equally important for evolutionary dynamics, but has received less attention. When populations are so small that drift overwhelms selection, deleterious mutations frequently fix [16,22,23], causing fitness to decline. If these populations are not all destined to become extinct, then as deleterious mutations are fixed, a fundamental change must occur in the distribution of mutational effects. Two possibilities have been proposed: that the accumulation of deleterious mutations is accompanied by an increase in the mean effect size of deleterious mutations (negative epistasis) or by an increase in the rate of beneficial mutations (compensatory epistasis) [16,22,23] (Figure 1). Both of these ideas are termed epistatic because the effect of a mutation, and thus the distribution of mutational effects, changes according the genetic background of the organism in which it appears.

We used experimental evolution of a bacteriophage to investigate how the distribution of beneficial and deleterious mutations changes (i.e., epistasis) when fitness changes in the course of evolution, and how this impacts adaptation. We corroborate the finding that in large populations, fitness increases to a plateau. We also report the complementary effect that in small populations, fitness does not decrease indefinitely, but reaches a lower plateau. The equilibrium level of fitness is thus largely determined by the effective population size, and we show that this is a consequence of simple changes in mutational effects that cause qualitative changes in the processes of selection and drift.

## Results

### Experimental Evolution of Fitness Plateaus

To study the impact of population size on adaptation, we used experimental evolution in the bacteriophage φX174, an organism in which population sizes can be controlled by adjusting the bottleneck size at transfer. We used phage populations to investigate two related questions: First, do populations evolve to a fitness equilibrium? Second, do populations that differ in effective population size reach different fitness equilibria? As a measure for fitness, we used competition with a reference strain [38]. Because there are few interactions between viral plaques during evolution, nontransitive fitness interactions are not expected to affect the outcome; thus competition assays with a common ancestor give an accurate assessment of fitness.

To identify fitness equilibria, we initiated experimental populations with either an ancestor of high fitness or an ancestor of low fitness. We then investigated whether the populations converged toward the same fitness value, irrespective of their starting condition. If populations that were initialized with high- or low-fitness ancestors converged in fitness during evolution, the evolved fitness levels of these populations were concluded to bracket a fitness equilibrium. The high-fitness ancestor was a clone derived from a phage line that had been passaged in our laboratory for 100 transfers at a large population size (≈10^4^). Two lower fitness ancestors were derived by serially bottlenecking this clone and selecting small plaques. This procedure resulted in two strains that had low fitness in competition with the ancestral strain.

**Figure 2:**
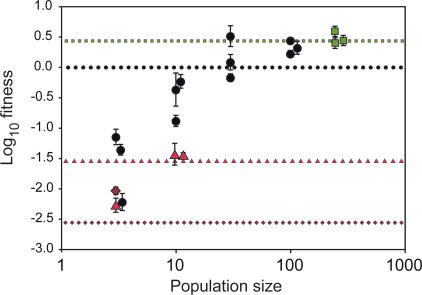
Changes in Mean Population Fitness Dotted lines indicate the fitness of ancestral clones: the high-fitness clone (black circles), lower fitness clones (bright red triangles and dark red diamonds), and the evolved highest fitness ancestor (green squares); this ancestor was derived from the population having the highest fitness after 90 transfers (population 100c). Each point indicates the average fitness of an evolved population, with error bars indicating one standard error. Some populations are slightly displaced on the *x*-axis for clarity. The population bottleneck sizes were three, ten, 30, 100, and 250. Five lineages were propagated at bottleneck sizes three and ten, and three lineages were propagated for all other bottleneck sizes. The color and shape of each point indicates the ancestral clone from which that population was derived. The fitness of the high-fitness ancestor was set equal to zero on a log_10_ scale, and all other fitness values are relative.

We initiated three populations from the high-fitness ancestor at each of four population sizes: three, ten, 30, and 100. We also initiated three populations from one low-fitness ancestor (two at a population size of ten and one at a population size of three, as well as one population from a very low-fitness ancestor, at a population size of three (Figure 2). During experimental evolution, all phage populations were subject to mutagenesis (see Materials and Methods).

After 90 transfers, the fitness of populations bottlenecked at sizes of three, ten, 30, and 100 were measured to test for convergence (Figure 2). We focus first on the ten populations bottlenecked at effective population sizes of three and ten, which exhibited evidence of convergence in fitness (Figure 2). Of these ten populations, all six populations that were initiated from a high-fitness ancestor declined significantly in fitness. Of the populations initiated from lower fitness ancestors, one increased in fitness (*N*_e_ = 3), two maintained almost constant fitness (*N*_e_ = 10), and one declined only slightly in fitness (*N*_e_ = 3) (Figure 1). In the populations bottlenecked at larger effective sizes (*N*_e_ = 30 and *N*_e_ = 100), five of the six evolved to a mean fitness above that of the high-fitness ancestor. It was thus difficult to ascertain whether these populations were evolving towards an equilibrium fitness. To establish whether these larger populations were evolving toward an equilibrium, we took the *N*_e_ = 100 population that had evolved the highest fitness and propagated it at an expanded population size of *N*_e_ = 250 for an additional 55 transfers (275 generations). Three *N*_e_ = 250 population replicates were propagated, and none changed significantly in fitness (Figure 2). This lack of change in fitness suggested that the *N*_e_ = 100 populations were at or near fitness equilibrium. Thus, as is illustrated in Figure 2, the trajectory of fitness change depends both on the effective population size and the initial fitness of the population. This suggests that changes in fitness lead to fundamental changes in the mutational distribution. Secondly, the final level of the fitness plateau appeared to depend explicitly on the population size; populations of different initial fitness converged toward similar endpoints.

Although care was taken to minimize the opportunity for cross-contamination between populations, it was important to directly rule out that contamination was responsible for the fitness convergence. To ascertain this, we sequenced the complete genome of one clone from each evolved population. Convergence of substitutions at a large fraction of sites would imply contamination. However, the number of sites at which convergence occurred was not significantly greater than expected by chance (see Materials and Methods), so cross-contamination was ruled out as a cause of fitness convergence.

The sequence data revealed that the fitness convergence was achieved despite little indication of genetic convergence. Hence our system reproduced one previously observed phenomenon in experimental evolution: fitness in a large, adapting population does reach a plateau with different starting genotypes. Interestingly, our results show that this effect can be extended to small population sizes: small populations converge to low-fitness equilibria without genetic convergence.

### Models Underlying Population Size–Dependent Fitness Plateaus

The presence of fitness equilibria has specific implications for how epistasis influences the rate and shape of the mutational distribution. In previous studies that have focused on the accumulation of deleterious mutations in small populations, two general hypotheses have been put forth that can prevent continuous fitness decline [16,17,22,23]. One hypothesis, commonly referred to as negative epistasis (synergistic epistasis in the case of deleterious mutations), postulates that the effect size of novel deleterious mutations increases as fitness decreases (i.e., as more deleterious mutations accumulate), and that this makes selection more efficient in removing these deleterious mutations [39] (Figure 1B). The second hypothesis, compensatory epistasis, suggests that some fraction of (formerly) deleterious mutations become beneficial (i.e., *compensatory*) when organismal fitness decreases (Figure 1C) and that this effect slows the fitness decline in small populations.

We focus on testing these two models here, by using an explicit model to describe how epistatic changes affect fitness convergence and equilibria. In this model, the deleterious and beneficial substitution rates (*k*_d_ and *k*_b_, respectively) change as fitness changes. For simplicity, we illustrate the case in which the effect on fitness of all beneficial mutations is identical, as is the effect on fitness of all deleterious mutations (in all later analyses, we use simulations in which mutational effects are modeled as a distribution). The substitution rates of deleterious and beneficial mutations are thus:

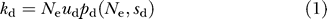


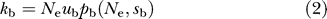
in which *N*_e_ is the population size, *u*_d_ is the deleterious mutation rate, *u*_b_ is the beneficial mutation rate, *p*_d_(*N*_e_,*s*_d_) = (1 − e^2*s*^)/(1 − e^2*Ns*^) [1] is the probability of fixation of a deleterious mutation of effect size *s*_d_, and *p*_b_(*N*_e_,*s*_b_) = (1 − e^−2*s*^)/(1 − e^−2*Ns*^) is the probability of fixation of a beneficial mutation of effect size *s*_b_. If a population is reduced in size such that fitness begins to decline, by definition,

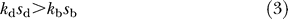
or

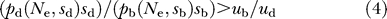



For the fitness decline to stop, Expression 4 must become an equality.

We can thus dichotomize the two epistatic hypotheses: The first hypothesis, negative epistasis, proposes that a decrease in the left side of Expression 4 occurs through an increase in the average selective coefficients, *s*_d_ and *s*_b_. The second hypothesis, compensatory epistasis, states that decreases in fitness cause an increase in the ratio between the beneficial mutation rate and the deleterious mutation rate, such that the right side of Expression 4 increases. We focus here on testing these two forms of epistasis, because they are the most commonly modeled forms. If there is either no epistasis or positive epistasis, then the fitness decline will continue unchecked, and no fitness equilibrium occurs. Similar logic applies to the case of large populations adapting to a constant environment. In the absence of epistatic effects, or if positive epistasis operates, fitness would increase continuously without reaching an equilibrium.

### Mutation Accumulation

**Figure 3:**
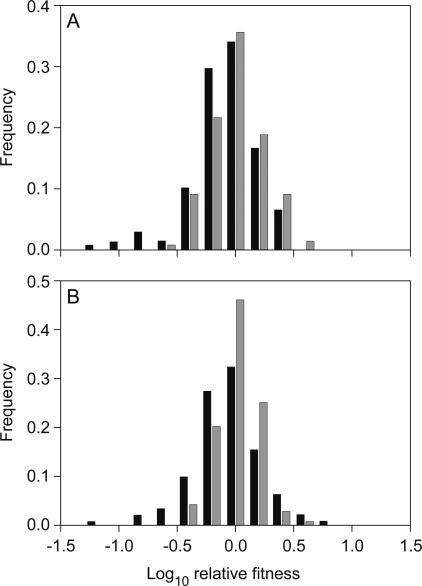
Mutation Accumulation (A) Fitness measurements of the high-fitness MA lines. The fitness values of the parents are shown in gray, the offspring in black. Note that all fitness values for each MA experiment were scaled such that the mean of the parental log_10_ fitness values was zero; this was done so that the error in fitness measurements was approximately normal, and so all three separate experiments in the low- and high-fitness lines could be combined on the same axis. (B) Fitness measurements of the low-fitness MA lines. Shading and scaling are the same as in (A). In both (A) and (B), a considerable shift to the left (lower fitness) can be observed after a single round of mutagenesis. In the low-fitness lines, several offspring clones with fitness greater than the parental clones can be observed. In the high-fitness lines, no such offspring clones are observed.

In order to understand which mechanism was responsible for the fitness equilibria we observed in our experimental populations, we needed to discriminate between the two main epistatic mechanisms (negative or compensatory epistasis). This required determining the ratio of beneficial and deleterious mutations, and the distribution of the effects of these mutations. To estimate these quantities, we used MA. In an MA experiment, mutations are allowed to accumulate by randomly collecting the progeny of a parental phage with no bias against low-fitness progeny. By measuring the fitness of a number of parents and a number of randomly chosen progeny, it is possible to estimate the fitness effects of mutations that occur in a single generation. We determined the fitness of 50 parents and 50 progeny for three high-fitness populations and three low-fitness populations (Figure 3; Materials and Methods). If negative epistasis is the primary mechanism promoting fitness equilibria, then we expect that mutations occurring in low-fitness populations will have larger selective effects than those occurring in high-fitness populations. If compensatory epistasis is the main mechanism operating, a higher proportion of the mutations occurring in low-fitness populations should be beneficial.

We used maximum-likelihood (ML) analysis [5] to estimate the distribution of mutational effects, *s,* and the proportion of beneficial mutations, which we refer to as *B,* and define as *u*_b_/(*u*_b_ + *u*_d_). Importantly, this analysis assumes that the distribution of mutational effects can be modeled as a reflected gamma distribution. The gamma distribution is used because it has great flexibility in terms of the shapes it can assume, with shapes ranging from highly leptokurtic to exponential to log-normal to Gaussian (Figure S2). However, the assumption of a reflected gamma distribution constrains beneficial and deleterious mutational to follow the same distribution. Additionally, the model is limited in its power to detect asymmetric epistasis between beneficial mutations (i.e., if only beneficial mutations increase in effect size as fitness decreases). However, such asymmetric epistasis does not seem very plausible. If the distribution of deleterious mutations does not depend on fitness, but the distribution of beneficial mutations does depend on fitness, then the fitness of an individual carrying one beneficial and one deleterious mutation would depend on the order in which the mutations occur, which is nonsensical. We discuss this idea in more detail in Text S1.

Estimating *s* and *B* requires an estimate of the mutation rate *(U)*. We derived this estimate from sequence data rather than the ML analysis to avoid conflating estimates of *s* and *U* [5]. We used the sequence data to determine the rate of synonymous substitutions, from which we inferred the mutation rate imposed under experimental evolution. There was a slight, but not significant, decrease in the rate of synonymous substitution in larger populations, suggesting that synonymous substitutions may have been slightly deleterious (unpublished data). We therefore used the rate of synonymous substitution observed in the populations bottlenecked at *N*_e_ = 3 and *N*_e_ = 10 to estimate the neutral mutation rate. (We expect that in these populations synonymous substitutions behaved neutrally, i.e., the selective coefficient is less than the inverse of the population size.) The average rate of synonymous substitution in these lineages was 0.18 per transfer. We extrapolated this rate to a genomic rate by multiplying by the ratio of total sites to synonymous sites in the ancestral genome while accounting for a proportion of lethal mutations (see Materials and Methods). The estimated genomic mutation rate was 0.56 per transfer.

**Figure 4:**
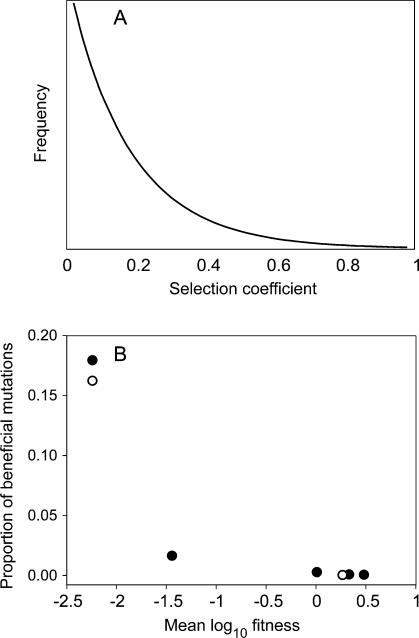
Mutational Parameters (A) Shape of the joint ML gamma distribution of mutational effects for all MA datasets. The distribution is extremely leptokurtic (L-shaped), with the majority of mutations having very small selection coefficients, although a significant proportion have large selection coefficients. (B) Relationship between fitness and rate of compensatory mutation. Filled circles indicate the inferred proportion of compensatory mutations using the ML gamma distribution (illustrated in [A]) and the experimental fitness equilibria (see Materials and Methods). Open circles indicate the ML estimation from the mutation accumulation data. The close match between the two methods suggests that the estimates of *B* are robust. The two overlapping points have been displaced slightly for clarity. It appears that there is a nonlinear relationship between the proportion of beneficial mutation and log-fitness. This is not the relationship that would be expected under the simplest hypothesis of compensatory mutation (see text for more details).

The ML estimate of the mean mutational effect over all populations had an expected value of 0.35 (0.082 per generation) (Figure 4A). The estimate of the mean effect was not different within the groups of low- or high-fitness populations (likelihood ratio test [LRT], χ^2^ 4 *df, p* = 0.21, χ^2^ 4 *df, p* = 0.22). Although the estimate was slightly lower in the high-fitness populations (0.29 as compared to 0.40), this difference was not significantly different between the two groups (χ^2^ 2 *df, p* = 0.49; Figure S1). The ML estimate of beta, which describes the shape of the distribution, was 1.0, and again, did not differ significantly between the two groups (Figure S1). This distribution of mutational effects agrees well with values reported for other viruses [40,41]. The ML estimate of *B,* the proportion of beneficial mutations, was zero for all high-fitness populations and greater than zero in all low-fitness populations. For two of the low-fitness populations, the model in which *B* was greater than zero gave a significantly better fit than the model in which *B* was constrained to zero (LRT, χ^2^ 1 *df, p* = 0.39, 0.010, and 0.0044 for these three ML analyses). The joint ML estimate of *B* for all low-fitness populations was 16%, and was not significantly different between these populations (LRT, χ^2^ 2 *df, p* = 0.63, Figure 4B). These results suggest that populations with high and low fitness may differ in the proportion of beneficial mutations, but not in the effect size of mutations. This provides our first support for compensatory epistasis.

We tested the reliability of our results by using a different method to estimate *B* for all the evolved populations. For a certain *N*_e_ and a specified distribution of *s,* it is possible to use numerical simulations to find the value of *B* at which mean population fitness no longer increases or decreases. Using the above estimate for the shape of the distribution of mutational effects, we could thus obtain an estimate of *B* at which each of the evolved populations should not increase or decrease in fitness (see Materials and Methods). Using this method to estimate *B* and a constant distribution of *s* (as was found using the maximum likelihood analysis of the MA data), suggested that *B* must increase to approximately 18% to maintain fitness equilibrium in the *N*_e_ = 3 populations. We used additional simulations to derive the value of *B* required for all other population sizes to maintain fitness equilibria. We then used the median values of the experimentally determined equilibrium fitness values at each *N*_e_ (Figure 2) to plot *B* as a function of fitness (Figure 4B). *B* increased in a nonlinear manner with fitness. For comparison, the two ML estimates of *B* from the MA data are also plotted in Figure 4B. The close match between the two methods suggests that the estimates of *B* are robust.

### Testing for the Effect of Lethal Mutations

The results of the MA experiment and the above analysis suggest that an increase in the ratio between the rates of beneficial and deleterious mutations *(B)* allows low-fitness populations to reach a fitness equilibrium. There are two different ways in which this ratio can increase, either through an increase in *u*_b_, the rate of beneficial mutations, or through a decrease in *u*_d_, the rate of deleterious mutations. The two alternatives have different biological meanings. An increase in *u*_b_ with decreasing fitness would correspond to the hypothesis of compensatory epistasis. A decrease in *u*_d_, the rate of deleterious mutations, could result if a large fraction of deleterious mutations become lethal in less fit genotypes. In our experimental regimes, the number of lethal mutations was not directly measured, and it was thus important to test for such an increase directly.

A change in the number of lethal mutations with changing fitness would manifest as a change in the genomic mutation rate, *U,* which is the sum of *u*_b_ and *u*_d_. We derived an independent measure of *U* based on the total number of nucleotide substitutions that occurred in all lines; we used a ML analysis to determine the *U* that best fit the observed number of substitutions in each population (see Materials and Methods). A model in which *U* varied across populations did not significantly improve the likelihood over a model with a single *U* (LRT, χ^2^ 15 *df, p* = 0.18). There is thus no evidence that low-fitness populations have lower *U,* as would be expected if the proportion of lethal mutations had increased in these populations. The new joint ML estimate of *U* using the nucleotide substitution data was 0.64. This closely matched our first estimate of 0.56, which relied only upon the number of synonymous substitutions observed in the *N*_e_ = 3 and *N*_e_ = 10 lines. Again, this suggests that our results are robust and internally consistent, and that increases in *u*_b_, and not a change in the genomic mutation rate, accounted for the observed changes in *B* (Figure 4B).

## Discussion

In the present paper, using experimental evolution of viruses, we have shown that populations tend to converge to a fitness equilibrium that depends on their population size. Large population–size lines converged to high fitness, and small population–size lines to low fitness (Figure 2). The observations of such fitness equilibria in all the experimental populations presented here, together with changes in the rate of adaptation observed in other experimental populations [32,42,43], demonstrate a simple, but perhaps underappreciated fact about the nature of mutational effects: they are dynamic. This means that mutational distributions are epistatic: the effect size, the sign (beneficial or deleterious), or both, depend on the genetic background of the organism in which they appear. Despite the evidence that the rate and distribution of mutation effects change as organismal fitness changes, a considerable amount of effort has been put toward obtaining precise estimates of these parameters [6,14,25–27,44–48], resulting in significant disagreement over what the relevant values may be [48,49]. We propose that a more careful examination of how these parameters are affected by organismal fitness may give greater insight into how adaptation occurs, and provide some resolution to the qualitatively different conclusions concerning the rate and shape of mutational distributions.

This distribution of mutational effects has significant implications for adaptation, because the direction and rate of fitness change in any population is governed by four quantities intrinsic to the organism: the rate and mutational distribution of beneficial mutations and the rate and mutational distribution of deleterious mutations. As shown above, that populations at a specific effective population size converge toward a single level of fitness necessarily constrains the values and dynamics of these quantities. In a population in which mean individual fitness is increasing, the rate of fixation of beneficial mutations multiplied by the mean effect size must outweigh this analogous quantity for deleterious mutations. In order for the rate of adaptation to decrease as fitness increases, one or more of the following must occur: the mean effect size of beneficial mutations must decrease; the rate of beneficial mutations must decrease (and consequently the rate of deleterious mutations must increase); or the effect size of deleterious mutations must decrease (although the consequence of this does depend on population size. When 1/*N*_e_ is less than the average mutational effect, a decrease in effect size will make deleterious mutations less visible to the purifying action of selection, and this effect will outweigh the fact that each deleterious fixation event has a lesser effect on fitness). Conversely, if a population is decreasing in fitness, to stop this decrease, one or more of the following must occur: the mean effect size of beneficial mutations must increase; the rate of beneficial mutations must increase (and consequently the rate of deleterious mutations must decrease); or the effect size of deleterious mutations must increase.

To untangle the different possibilities, we performed MA experiments on lines differing by approximately 300-fold in fitness to characterize the distribution of deleterious mutation effects on fitness (Figure 4). This distribution of mutational effects is leptokurtic, with most mutations being of small effect, and a few having large effects (Figure 4A). Across a very wide range of fitness values, this distribution of effects appears to remain approximately constant (Figure S1). Additionally, the shape of the distribution is strongly supported by the genomic data, which show that even when populations are maintained at large population sizes and fitness does not change, substitutions continue to accrue; these must be of very small effect. However, if all mutations were of small effect, they should be immune to selection in small populations. This was not observed; both deleterious and beneficial mutations were subject to selective forces, even in the smallest of the populations (*N*_e_ = 3). Although the distribution differs slightly from others that have been recently proposed, it concurs well with others [41]. We also note that the distribution we have estimated includes both synonymous and non-synonymous mutations, and that this also contributes to the leptokurtic shape.

The deleterious mutation distributions found do not provide significant support for the hypothesis that the effect size of deleterious mutations changes with fitness (i.e., positive or negative epistasis between deleterious mutations) (Figure 1), despite the fact that clones differing by a 300-fold factor in fitness were tested. Although there is some suggestion that mutational effects increase slightly in low-fitness populations (negative epistasis), this effect did not approach significance. Additionally, the size of the effect was not large enough to account for the evolution of fitness equilibria that were observed. This conclusion of constant mean mutational effects is further supported by the molecular data, which show that populations maintained at or near fitness equilibrium have the same, and possibly greater, rates of substitution when compared to populations experiencing declines in fitness. This outcome is precisely the opposite of that expected if equilibria were maintained through more efficient selection (i.e., negative epistasis, or a larger effect size of deleterious mutations). This type of epistasis would act to purge more, not fewer, substitutions from the population.

The absence of any change in the effect size of deleterious mutations suggests that a significant change must occur in either the rate or effect size of beneficial mutations. We provided strong support for compensatory epistasis using a ML analysis of MA data. We also showed that the increase in *B,* the proportion in beneficial mutations, is not driven by a decrease in the deleterious mutation rate (i.e., an increase in the nonviable mutation rate). This is shown through an analysis of the molecular data and is also suggested by the magnitude of change: it is likely that the beneficial mutation rate changes by much greater than 10-fold between the high- and low-fitness lines. A comparable change in the overall mutation rate would be easily detected; indeed, it is very unlikely that the rate of lethal mutation in the low-fitness lines increased more than 2-fold (see Materials and Methods).

The epistasis we have observed in our data is a result of a change in the probability that a given mutation is beneficial or deleterious and is dependent on the fitness of the organism in which it appears. The shape of the distribution of beneficial and deleterious effects remains approximately the same. This type of epistasis has been studied less often than other forms, but is consistent with several experimental estimations of epistasis that have focused only on deleterious mutations. The positive epistasis found to be operating between deleterious mutations in several recent studies [40,50,51] could be due to the presence of compensatory mutation. Moreover, several recent comparative studies have emphasized the importance of compensatory epistasis [52–55] in evolution. Together these results encourage reconsidering classic models to take compensatory epistasis into account. We must also emphasize that the model presented here is based on statistical properties of the distribution of mutational effects, and only makes predictions about the expected values of mutations. Thus we do not expect that all individual mutational interactions will conform to the model; there is already good empirical evidence for other types of epistatic interactions between mutations (e.g., antagonistic epistasis [56]) .

Additionally, it is important to note that the model of compensatory epistasis suggested here is not subsumed by any of the simpler models of epistasis (Figure 1) (although similar dynamics can be observed in some more complex models of adaptation [57]). All of the simpler models of epistasis are defined according to deviations from expected fitness when there are combinations of either deleterious or beneficial mutations, and therefore involve a change in the mean of the distribution of deleterious *or* beneficial effects (Figure 1). The epistasis we have observed here relies simply on the change from beneficial to deleterious (and vice versa) of a given mutation according to the genetic background and fitness of the organism in which it appears; the mean of the distribution of beneficial and deleterious effects remains the same (Figure S1). Compensatory epistasis also suggests that few mutations are strictly deleterious or beneficial; they are *conditionally* deleterious or beneficial.

The nonlinear relationship that we observed between *B* (the proportion of beneficial mutations) and log-fitness is noteworthy. Because the shape of the mutational distribution does not change with fitness, log-fitness should, on average, scale linearly with the number of deleterious mutations that an individual has. Under the simplest mutational model, deleterious mutations interact multiplicatively (with *i* deleterious mutations, *W* = (1 − *s*)*^i^*), and each deleterious mutation would result in a single beneficial mutation becoming available—the back mutation. Because the expected value of log-fitness and the number of deleterious mutations scales linearly (log(*W*) = i*log(1 − *s*)), and the number of deleterious mutations scales linearly with the number of beneficial mutations (in a 1:1 manner), then log-fitness should also scale linearly with the beneficial mutation rate. This is true under any model in which each deleterious mutation results in a constant average number of newly available beneficial mutations. The nonlinearity of the relationship between *B* and fitness thus implies that the number of beneficial (compensatory) mutations that become available in the context of each additional deleterious mutation increases as fitness decreases. This suggests that there may be changes in the number of loci involved in compensatory mechanisms as fitness declines. One interpretation of this is that pleiotropic interactions between genetic loci (functions) increase as fitness decreases. Although a negative relationship between the rate of compensatory mutation and fitness has been predicted by modeling, little has been predicted concerning pleiotropy. We hope that our results will spur new interest in this topic.

This study is the first to propose and test an explicit model of adaptation in which organismal fitness specifies both the rate and distribution of deleterious and beneficial mutations. Although the model necessarily relies upon an idealized form of the genotypic landscape, it presents specific and testable predictions of the circumstances under which populations will increase or decrease in fitness.

Additionally, the model offers a simple mechanistic explanation for the diminishing rate of fitness increase observed within large populations adapting in a constant environment [24], as well as for the prevention of continued fitness degradation in small populations that have accumulated deleterious mutations. Finally, these data suggest that attempts to measure the rate (or distribution) of mutational effects must take into account that these rates may change by orders of magnitude as the fitness of an organism increases or decreases.

## Materials and Methods

### Strains.

The original φX174 bacteriophage used in this study was derived from a clone kindly provided by B. Fane. This clone was passaged for 100 transfers at 32 °C to allow general adaptation to laboratory conditions. The deleterious mutants were obtained through serial bottlenecks with mutagenesis (see below), during which small plaques were purposefully chosen. The host was Escherichia coli (mutT), obtained from D. Bregon at INSERM.

### Passaging.

During passaging, phage were mutagenized in 250 mM hydroxylamine (HA) [58], 1 mM EDTA at 37 °C for 140 min. Mutagenesis limited back mutation, prevented the mutation rate from evolving [59], and allowed simplifications to be made in modeling mutation. Mutagenic treatment was stopped by 100- to 1,000-fold dilution into Luria-Bertani media with 0.5× NaCl (LC media). The phage were then plated on LC agar plates with 3 ml of soft agar and 0.3 ml of E. coli (mutT), and then grown overnight at 32 °C. From these plates, a number of plaques equal to the bottleneck size were randomly selected and diluted into culture tubes containing 3 ml of 1 mM EDTA. These tubes were vortexed and centrifuged, after which 0.5 ml was removed to a fresh Eppendorf tube. Chloroform was added, the tubes were vortexed and centrifuged, and 0.3 ml was removed. This was used for further mutagenesis, and the remainder of the stock was stored in 40% glycerol at −20 °C.

### Competition experiments.

Competition experiments were designed and performed as described previously in Burch and Chao [38]. Briefly, each strain was mixed in an approximately 1:1 ratio with a marked ancestral strain. This mixture was then plated twice to obtain a precise measure of the starting ratio of the two strains. At least three competition plates were plated for each strain (except when assessing the fitness of the mutation accumulation lines, for which only one plate was used); each of these were grown for 18 h (the time allowed for growth during experimental evolution), after which the phage from each plate were harvested and re-plated once to find an ending ratio for the two strains.

### Mutation accumulation.

In an MA design, mutations are allowed to accumulate by randomly collecting the progeny of a parental phage with no bias against low-fitness progeny. The high-fitness parents were derived from two of the *N*_e_ = 100 populations (Figure 3A), and the low-fitness parents from two of the *N*_e_ = 3 populations (Figure 3B), after fitness convergence. To ensure that changes in mutational parameters were not due to the particularities of one clone, populations were also used, one from a low-fitness line and one from a high-fitness line, for a total of six separate MA experiments. Mutagenesis was performed in the same manner as during passaging. Fifty progeny clones were chosen randomly without respect to fitness. The fitness of these clones was measured using competition experiments, as well as the fitness of 50 parental clones.

### Maximum likelihood model.

The ML model used models the distribution of selective coefficients *(s)* as a reflected gamma distribution [5,45]. Although this model can simultaneously estimate *U,* the distribution of *s,* and *B,* we fixed *U* based on the estimate from the synonymous substitution rates in the small bottleneck lines (see below). This independent estimate of *U* allowed greater confidence in the estimates of the distribution of *s* and *B.* The likelihood analysis assumes a normally distributed measurement error; the estimate of this error was allowed to vary between experiments, because all six MA experiments were not conducted simultaneously (although the fitness measurements of all ancestral and descendent clones were performed simultaneously). For the analysis, log-transformed values of fitness were used, because without this transformation, error was not normally distributed. Additionally, the means of the ancestral (parental) generation were set to one (zero on a log scale) for the analysis. After the analysis, the parameters were used to calculate a gamma distribution, and this distribution was then re-transformed to obtain the standard mutational distribution (transformation: *s* = 1 − 10^−*s*^). The maximum likelihood shape and size parameters for the mutational distribution values were 5.3 and 1.0, respectively.

The likelihood surfaces were generated by calculating the likelihood values over a grid of parameter values while maintaining the other parameters at their ML-estimated values (for *B,* 0 in the high-fitness lines and 0.16 in the low-fitness lines).

### Sequencing, alignment, and sequence analysis.

Some sequencing reactions were performed using the BigDye Terminator v3.1 kit and analyzed using an ABI sequencer; others were performed by the University of California San Diego Cancer Center or at INSERM. Alignments were done using Sequencher. Because φX174 contains multiple overlapping reading frames, sequence analysis was done using software written in C or Perl.

### Estimation of the mutation rate.

The simplest method of estimating the mutation rate is to estimate the rate of mutation in a theoretically neutral class of sites, and then to extrapolate this rate to the entire genome. However, two problems arise in using such an analysis. Firstly, the mutagenesis resulted in a significant bias toward cytosine → thymine transitions: of all observed substitutions, 89% were of this nature. Additionally, a significant number of mutations are either lethal or highly deleterious, and such mutations will never occur in the population during evolution. It is very likely that the majority of such mutations are missense or nonsense substitutions. Because the ML model of the mutational distribution can only account for a unimodal distribution of mutations, directly extrapolating the genome-wide mutation rate from the rate at synonymous sites would result in an overestimation of the mutation rate, because this rate would include all missense and nonsense mutations that lead to nonviable phenotypes. Therefore we used the rate of synonymous substitution, as well as information about the specific nature of mutations (missense or nonsense) and the amount of over-dispersion of substitutions to infer an *effective* genomic mutation rate. This effective genomic mutation rate only includes mutations that could actually be introduced into the evolving population. This mutation rate is thus the mutation rate for viable phage, which we define as those phage that are both capable of replicating and of forming visible plaques.

We base the estimate of the mutation rate on the substitutions observed in the populations propagated at the smallest effective population sizes, because mutations appearing in these lineages should be least subject to selection. Within these lineages, a total of 162 synonymous or intergenic cytosine → thymine substitutions occurred at 117 different sites. These substitutions are slightly over-dispersed when compared to the random expectation, although not significantly. If we assume that some proportion, λ_S_, of the synonymous cytosine → thymine transitions result in a nonviable phenotype, the over-dispersion is decreased. We fit the λ_S_ parameter by minimizing the χ^2^ goodness-of-fit statistic; the value of this statistic is approximately equal to the probability of observing the data. We estimate λ_S_ = 0.14, with 95% confidence limits ranging from λ_S_ = 0 to λ_S_ = 0.40. A similar analysis may be done with non-synonymous missense sites. During experimental evolution, a total of 141 missense cytosine → thymine substitutions at 120 different sites occurred. We removed one of these sites from the analysis, because the substitution occurred in more than four independent lines, and was thus likely subject to strong positive selection. The estimate of λ_M_ was 0.37, with 95% confidence limits ranging from λ_M_ = 0.01 to λ_M_ = 0.61. Finally, we examine nonsense mutations. Of the 560 substitutions observed over all lines during the period of evolution, only one was a nonsense mutation, although the expected frequency is 9%. We thus estimate that λ_N_ is essentially 1. All of these estimates are similar to previously estimates values in other viruses, with λ_S_ estimated at 0.11 and λ_M_ at 0.40 [41].

We calculated the effective mutation rate using these parameters. The genome of the ancestral φX174 clone contains 1,151 cytosines; at 309 (27%) of these residues, a transition from cytosine to thymine is synonymous or intergenic; at 736 (64%), a transition is non-synonymous; and at 106 (9%), a transition results in a change to or from a stop codon. The observed rate of synonymous cytosine → thymine substitution was 0.18 per transfer. With a total of 309 cytosines at which this transition is synonymous or intergenic, and an estimated λ_S_ = 0.14**,** this suggests that substitutions can occur at 265 of these sites. Similarly, with 736 cytosines at which cytosine → thymine substitutions are non-synonymous, and an estimated λ_N_ = 0**.**37, this suggests that substitutions can occur at 466 of these sites. The ratio of *total* substitutable cytosine sites to substitutable synonymous sites is 2.76**,** implying a genomic cytosine → thymine mutation rate of 0.50 per transfer. Finally, because cytosine → thymine substitutions account for only 89% of all substitutions, we estimate the total effective genomic mutation rate as 0.56 per transfer.

### Cross-contamination of experimental lines.

If cross-contamination occurred, one would expect that two lines would share substitutions at a large number of sites (thus significantly increasing over-dispersion). This is clearly not the case for synonymous substitutions. (There is no significant over-dispersion; the 95% confidence interval for λ_S_ includes zero.)

### Simulations.

We modified an individual-based model of adaptation [59] for the simulations. For all simulations, the populations were simulated using plaques (not phages) as the individuals. The justification for this simplification is that mutational variance is input into the population at a single stage during the plaque lifetime, after which the plaque develops into an adult with phage offspring that are almost genetically identical to the parent phage (the background rate of genomic mutation in the phage is approximately 10^−2^, so almost 99% of the phage within each plaque are genetically identical). Thus the bottleneck population sizes are an accurate characterization of the effective population size of the phage populations. Secondly, even if individual phage were tracked, this would give rise to similar rates of fixation (see Text S1). The two parameters of the distribution of mutation effects were calculated for four *U* values using the previously described joint ML approach. For intermediate values of *U,* the two parameters were extrapolated (power law fit, *r* ^2^ > 0.98), and the following equation was numerically solved for each population size:

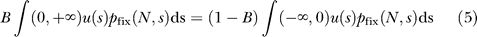
in which *B* is the fraction of beneficial mutations, u(s) is the ML gamma distribution of mutational effects, and *p*_fix_(*N,s*) is the probability of fixing a mutation of effect *s* in a haploid population of size *N:* (1 − e^−2*s*^)/(1 − e^−2*Ns*^) [1].

The relationship between log fitness and *B* was calculated using the mean equilibrium fitness of each population size and assuming a hyperbolic relationship, which implies the existence of a maximum fitness and a maximum value of 50% for *B.* For each value of *U,* the simulations took into account population size, initial fitness, and number of transfers. At least 1,000 simulations were run for each set of parameters, and the total number of mutations in each individual in the population was recorded. Likelihoods were calculated as the probability of picking an individual with the same number of fixed mutations as the number observed.

## Supporting Information

Figure S1 Confidence Limits for the Parameters of the Mutational Distributions in Low- and High-Fitness Clones When *B* Is Held Constant (A) Confidence limits for the ML parameter values of the gamma distribution in the low-fitness clones. The proportion of beneficial mutations, *B,* was left at its ML value of 0.16. The joint likelihood surface for the three MA analyses in the low-fitness lines is shown.
(B) Confidence limits for the ML parameter values of the gamma distribution in the high-fitness lines. The proportion of beneficial mutations, *B,* was left at its ML value of zero.
(C) Confidence limits for the ML parameter values of the gamma distribution in all MA lines. The proportion of beneficial mutations, *B,* was left at its ML value (0.16 or 0). The joint log-likelihood value is indicated to the right of each panel; the ellipses indicate the 50%, 80%, 90%, and 95% confidence limits. The *y*-axis shows the estimated mean of the distribution, while the *x*-axis is the coefficient of variation (1/√β). The estimated mean of the distribution is relatively constant for the two groups, providing little support for any hypothesis under which the mean value of the selection coefficient changes with fitness. However, there is less confidence in the shape of the distribution (beta).
(146 KB PDF)

Figure S2 Confidence Limits for the Parameters of the Mutational Distributions in Low- and High-Fitness Clones When Beta Is Held Constant (A) Confidence limits for the ML parameter values of the gamma distribution in the low-fitness clones. The value of beta was kept constant, at one.
(B) Confidence limits for the maximum likelihood parameter values of the gamma distribution in the high-fitness lines. Again, the value of beta was kept constant, at one. The *y*-axis shows the estimated mean of the distribution, while the *x*-axis indicates the estimated proportion of beneficial mutations *(B).*
(100 KB PDF)

Text S1 Asymmetric Epistasis (26 KB DOC)

### Accession Numbers

The GenBank (http://www.ncbi.nlm.nih.gov/Genbank) accession numbers for the genome sequences of the bacteriophage φX174 lineages discussed in this paper are EF380009—EF380032.

## Abbreviations

LRT: likelihood ratio test
MA: mutation accumulation
ML: maximum likelihood
