# Supplementary material for: Understanding the Evolutionary Fate of Finite Populations: The Dynamics of Mutational Effects
Source: PLoS Biol. 2007 Apr 3;5(4):e94. doi: 10.1371/journal.pbio.0050094 (PMC1845161; doi:10.1371/journal.pbio.0050094)
Supplement: Supplementary file 1 [file pbio.0050094.sg001.pdf]

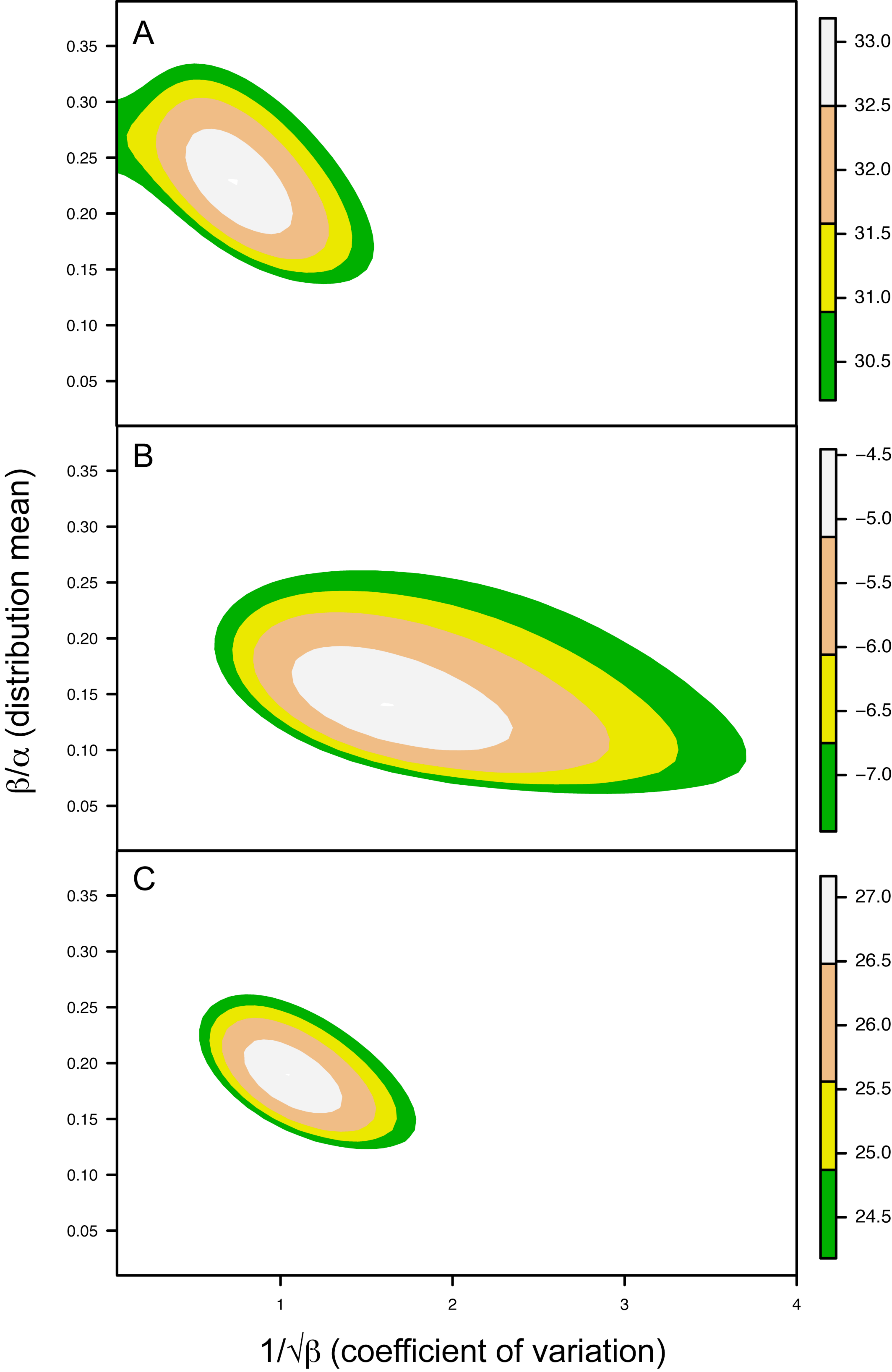
A
B
C
0.35
0.30
0.25
0.20
0.15
0.10
0.05
33.0
32.5
32.0
31.5
31.0
30.5
−4.5
−5.0
−5.5
−6.0
−6.5
−7.0
27.0
26.5
26.0
25.5
25.0
24.5
β/α (distribution mean)
1
2
3
4
1/√β (coefficient of variation)
