# Supplementary material for: Understanding the Evolutionary Fate of Finite Populations: The Dynamics of Mutational Effects
Source: PLoS Biol. 2007 Apr 3;5(4):e94. doi: 10.1371/journal.pbio.0050094 (PMC1845161; doi:10.1371/journal.pbio.0050094)
Supplement: Supplementary file 2 [file pbio.0050094.sg002.pdf]

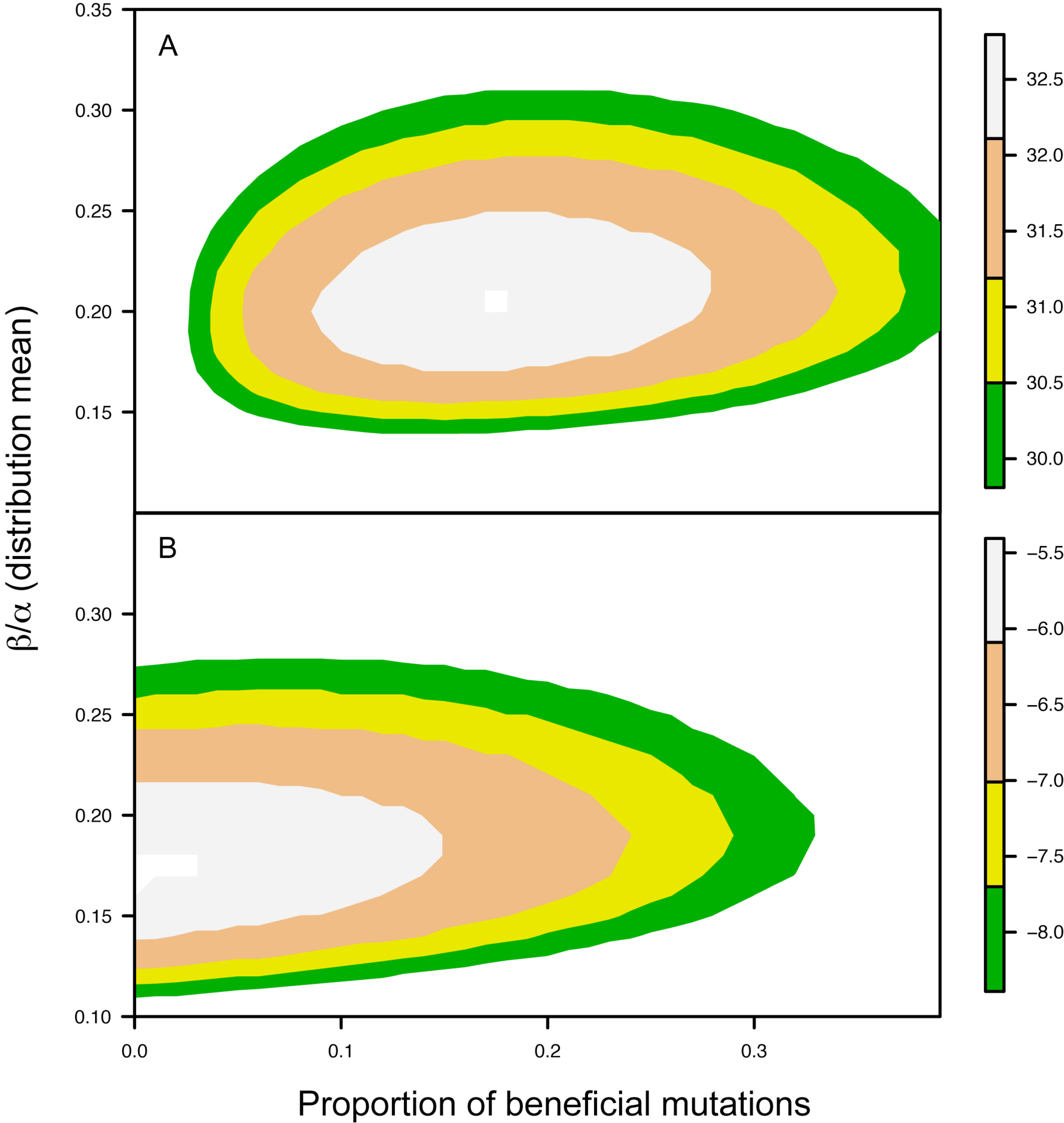

A
B
0.35
0.30
0.25
0.20
0.15
0.10
0.0
0.1
0.2
0.3
β/α (distribution mean)
Proportion of beneficial mutations
32.5
32.0
31.5
31.0
30.5
30.0
−5.5
−6.0
−6.5
−7.0
−7.5
−8.0
