# Supplementary material for: Understanding the Evolutionary Fate of Finite Populations: The Dynamics of Mutational Effects
Source: PLoS Biol. 2007 Apr 3;5(4):e94. doi: 10.1371/journal.pbio.0050094 (PMC1845161; doi:10.1371/journal.pbio.0050094)
Supplement: Supplementary file 3 [file pbio.0050094.sd001.doc]

**Supplementary Text S1**

**Asymmetric Epistasis**

Our results suggest that the effect size of deleterious mutations remained unchanged over a 300-fold fitness change, and thus during further analysis we assumed that the effects of beneficial mutations were also constant, and that only their rate changed. Nevertheless, it is possible that the genetic landscape is constructed such that while the mean effect size of deleterious mutations does not change with fitness, the mean effect size of beneficial mutations declines as fitness increases. We show here using a simple genetic model that from a general standpoint**,** a genetic landscape in which mutations keep their beneficial or deleterious status (i.e. the relative rates do not change), but in which the effect of beneficial mutation decreases with fitness while deleterious effects remain constant, is not possible.

Consider a locus (e.g. an amino acid site) at which two types of mutation are available. Several are deleterious with effect *d* (constant with regard to fitness), and one is beneficial with effect *b(w)*, a decreasing function of fitness. If a deleterious mutation occurs first, then the fitness is *w_0_*·(1-*d*) and the fitness of the double mutant is

*w_0_*·(1-*d*)·[1+*b*(*w_0_*·(1-*d*))]	(6)

If the beneficial mutation occurs first, then fitness of the double mutant is

*w_0_*·(1+*b*(*w_0_*))·(1-*d*)	(7)

The two genotypes are identical, and thus should have the same fitness. Therefore:

*w_0_*·(1-*d*)·[1+*b*(*w_0_*·(1-*d*))] = *w_0_*·(1+*b*(*w_0_*))·(1-*d*)	(8)

and *b*(*w_0_*·(1-*d*)) = *b*(*w_0_*), which is only possible if *b*(*w_0_*) is constant and there is no change in the effect of beneficial mutation. It is similarly true that assuming negative epistasis between beneficial mutations necessitates negative epistasis between deleterious mutations.

The fact that we cannot detect beneficial mutations during mutation accumulation in high fitness lines implies that either their effect size is too small, or that they are too rare. However, if it is true that they have declined in effect size, then the above analysis suggests that deleterious mutations should have declined in effect size to an equal extent. As this was not the case we can reject the idea that only the effect size of beneficial mutations changes with fitness. Hence our model strongly suggests that compensatory epistasis is the key feature of the adaptive landscape and that any adaptive landscape that is compatible with our observations must contain substantial changes in the beneficial and deleterious status of specific mutations.

This simple model also demonstrates that when both beneficial and deleterious mutations are considered at different positions in the fitness landscape, the model of epistasis used for beneficial and deleterious mutations should be consistent with one another, as a beneficial mutation in one genetic background is a deleterious mutation in another genetic background. The above analysis suggests that when both beneficial and deleterious mutations are being considered, there is some logical inconsistency in presuming that epistatic interactions are antagonistic or synergistic.

**Plaque- versus phage-level selection**

At the plaque level, the fixation probability of a mutant is proportional to *N_p_⋅s_p_⋅P_p_* (where *N_p_* is the effective population size (number of plaques), *s_p_* is the selective advantage per period of plaque growth, and *P_p_* = 1/*N_p_* is the frequency of the mutant). Scaling per generation, if there are *g* generations in a plaque then the per generation parameters are *N_g_* ~ *g⋅N_p_* and (1+*s_g_*)^g^ = 1+*s_p_*, in which *N_g_* is the effective population size taking into account phage growth within a plaque, *s_g_* is the selective advantage per generation, and as the vast majority of mutation occurs during mutagenesis, *P_g_* = *P_p_*. As ln(1+*s*) ~ *s* for small *s*,

*g⋅s_g_* ~ *s_p_*	(1)

Substituting yields

*N_p_⋅s_p_⋅P_p_* ~ (*N_g_/g)*⋅(g*⋅s_g_*)*⋅P_g_*	(2)

and simplifying

*N_p_⋅s_p_⋅P_p_* ~ *N_g_⋅s_g_⋅P_g_*	(3)

and thus regardless of whether plaques or individual phages are considered, the probability (and rates) of fixation are very similar.
